# Immunomodulation of Patients with Canine Visceral Leishmaniasis at Different Stages: A 12-Month Follow-Up Study Using LaSap

**DOI:** 10.3390/vaccines13090933

**Published:** 2025-09-01

**Authors:** Kelvinson Fernandes Viana, Adrieli Barboza de Souza, Sara Torres, Maria Camila Escobar Garcia, Açucena Veleh Rivas, Alex Sander Rodrigues Cangussu, Francisca Hildemagna Guedes da Silva, Rodolfo Cordeiro Giunchetti

**Affiliations:** 1 Vaccine Development Technology Laboratory, Federal University for Latin American Integration (UNILA), Foz do Iguaçu 85870-650, PR, Brazil; drika.barbozas@gmail.com (A.B.d.S.); sara.torres@aluno.unila.edu.br (S.T.); mce.garcia.2016@aluno.unila.edu.br (M.C.E.G.); acucenarivas@gmail.com (A.V.R.); 2 Vaccine Laboratory, Federal University of Tocantins (UFT), Gurupi 77410-530, TO, Brazil; alexcangussu@mail.uft.edu.br; 3 Leopoldo de Meis Institute of Medical Biochemistry, National Institute of Science and Technology for Structural Biology and Bioimaging, National Center of Nuclear Magnetic Resonance Jiri Jonas, Federal University of Rio de Janeiro, Rio de Janeiro 21941-599, RJ, Brazil; hildemagnaguedes@gmail.com; 4 Cellular Interaction Biology Laboratory, Department of Morphology, Federal University of Minas Gerais (UFMG), Belo Horizonte 31270-901, MG, Brazil; giunchetti@gmail.com

**Keywords:** immunotherapy, canine visceral leishmaniasis, LaSap

## Abstract

**Background/Objectives**: Canine visceral leishmaniasis (CVL) is one of the main neglected protozoan diseases in the world. Dogs play a fundamental role in the maintenance of *Leishmania infantum* in the Americas, and we have already encountered resistance problems with drugs currently used in these animals. **Methods**: In view of this, two new immunotherapeutic protocols were tested in 48 dogs, using *L. amazonensis* antigens plus saponin (LaSap) and only *L. amazonensis* antigens (La) as a control group. Dogs naturally infected with *L. infantum* were divided into four groups, according to clinical staging. A total of 24 dogs (stages 1 and 2) received a four-dose protocol, and another 24 dogs (stages 3 to 5) received six doses. All animals received a booster dose every three months until they were one year old. **Results**: Our results showed that dogs in the early stages of the disease respond better and are able to remain stable for longer, maintaining baseline laboratory biomarkers, in addition to having a lower parasite load. **Conclusions**: On the other hand, dogs in more advanced stages have a poor response, with stage 3 being a key point in clinical progression or regression.

## 1. Introduction

According to the World Health Organization (WHO), leishmaniasis is a complex of neglected diseases caused by protozoa of the genus *Leishmania* sp. [1]. Insects of the genus *Lutzomyia* and *Phlebotomus* are the transmitters of the parasite in the New and Old World, respectively [2]. Widely distributed throughout the world, leishmaniasis is mainly related to areas with worse socioeconomic conditions [3]. In 2023, the WHO announced that Bangladesh had eliminated visceral leishmaniasis (VL) as a public health problem, the first country in the world to do so. This status was validated after Bangladesh reported less than 1 case per 10,000 people for the previous 3 consecutive years. This result was achieved through a series of factors associated with disease control [4].

VL is one of the most prevalent human diseases, with more than 20 *Leishmania* species reported worldwide [3]. The protozoan disease can be classified as anthroponotic (AVL) or zoonotic (ZVL), depending on the different vulnerable species involved [5]. In the context of ZVL, canine visceral leishmaniasis (CVL) is strongly linked to human cases. Furthermore, not only symptomatic dogs but also asymptomatic ones can act as a source of infection for the main phlebotomine vector, *Lutzomyia longipalpis*, in Latin America [6,7].

In both Brazil and Europe, dogs infected with *L. infantum* must be treated or euthanized. However, there are marked differences in the vector’s seasonality and infection pressure between the two regions. The monthly distribution and abundance of sand flies are influenced by biotic and abiotic factors. In addition, temperature, humidity, and precipitation influence the populations of these vectors [8,9]. These factors directly interfere with the treatment of dogs, and it is common for these animals to undergo new protocols with miltefosine at increasingly shorter intervals, directly impacting parasite resistance.

Immunotherapy as a treatment proposal for leishmaniasis has been a promising alternative for decades. Different researchers have used this treatment approach in humans and dogs, obtaining very encouraging results [10,11,12]. Our research group has been working for some years with total antigens of *L. amazonensis* associated with saponin as an alternative in the clinical management of canine patients with VL. However, the challenging complexity of this multifaceted disease makes us understand that dogs in different clinical stages may require different immunotherapeutic protocols. This particularity may be necessary because the canine patient requires more or less antigenic stimulation to remain clinically stable [13,14]. Therefore, in the present study, we present an analysis using two protocols in dogs with different forms of VL.

## 2. Materials and Methods

### 2.1. Animals

This study involved 48 dogs (24 males and 24 females) of different breeds, most of which were mixed breeds. Their ages ranged from 8 months to 10 years. All animals were naturally infected with *L. infantum* (diagnosed by serology and PCR) and staged according to the Brazilian and Leishvet guidelines. All animals underwent a battery of clinical, hematological (blood count), biochemical (AST, ALT, urea, and creatinine), and molecular (bone marrow qPCR) tests to establish the clinical staging of each patient. All dogs were from owners in the city of Foz do Iguaçu, Paraná, Brazil. The study was approved by the Ethics Committee for the Use of Animals (CEUA-UNILA), protocol 003/2022, on 8 April 2022.

### 2.2. Inclusion and Exclusion Criteria and Randomization

Only dogs that tested positive for *L. infantum*—based on laboratory diagnostics using ELISA (Biogene^®^, Recife, Pernambucio, Brazil), indirect immunofluorescence (Immunodot^®^, Jaboticabal, São Paulo, Brazil), and qPCR of bone marrow—were included in this clinical study. In addition, all owners agreed to sign a free and informed consent form. All blood samples from the dogs underwent serological and molecular tests to detect hemoparasitosis such as *Babesia*, *Ehrlichia* and *Anaplasma*. Animals that tested positive in any of the tests were treated before starting the study.

Dogs that presented any serious adverse reaction (e.g., seizure or anaphylaxis) after the application of immunotherapy would be monitored by the responsible clinical veterinarian and excluded from the study. Dogs that presented any other clinical pathological manifestation throughout the study, such that the therapeutic conduct could interfere with the results, would be excluded from the experiment. The animals were randomized using “https://www.random.org/”.

### 2.3. Experimental Design

All dogs were clinically staged between grades 1 and 5 and randomized into four groups, each containing 12 dogs. The study was divided into two treatment protocols: dogs in stages 1 and 2 received the four-dose immunotherapy protocol containing 100 micrograms of total *L. amazonensis* antigens (MHOM/BR/1977/LTB0016) plus 100 micrograms of saponin subcutaneously at 21-day intervals (LaSap). The control group also consisted of 12 dogs that received only 100 micrograms of total *L. amazonensis* antigens (La) subcutaneously at 21-day intervals. After the protocol ended, all dogs in both groups received a booster dose of their respective formulations every three months, until they completed 12 months.

**Figure 1:**
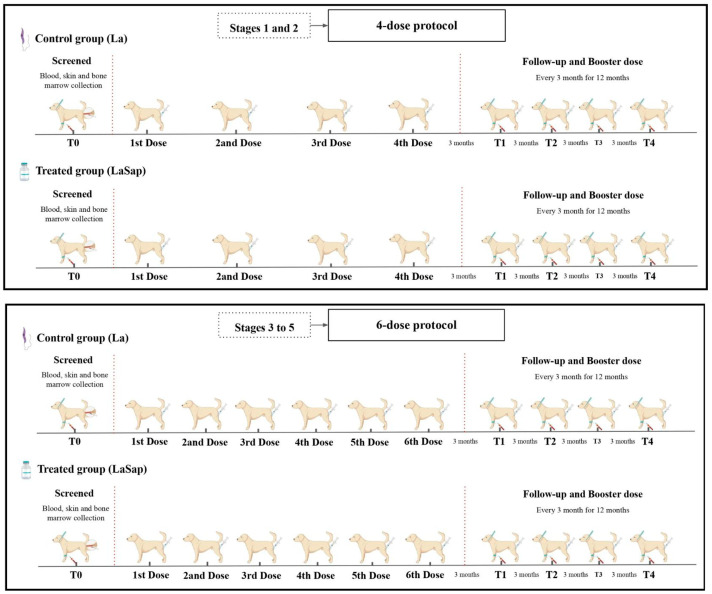
Experimental design using LaSap: Dogs in stages 1 and 2 received four doses at 21-day intervals via the subcutaneous route. Dogs in stages 3 to 5 received two initial doses at 7-day intervals; the third dose 15 days after the second dose; and the last three doses at 30-day intervals each. After this, all dogs received a booster dose every three months for one year. Time zero was 15 days before starting the experiment.

The second protocol consisted of dogs in clinical stages between grades 3 and 5. There were two groups (LaSap and La), with 12 dogs per group. In the LaSap group, all dogs received six doses of immunotherapy, with the first two doses 7 days apart, the third dose 15 days after the second, and the last three doses 30 days apart. The control group (La) received the same protocol, but without saponin in the formulation (Figure 1). After the protocol ended, the animals received a booster dose every 3 months for 12 months. Blood samples were collected from the animals at a specific time point (15 days before the start of the experiments) and on the day of each immunotherapy booster. The samples were used for hematological, biochemical, serological, and molecular analyses (zero time).

### 2.4. Hematology and Biochemistry

The hematological profile was analyzed by blood cell counts using an electronic hematology particle counter (BC2800Vet, Mindray, Hamburg, Germany). Differential leukocyte counting was performed on Giemsa-stained blood smears, and a total of 100 cells were counted. The biochemical evaluations consisted of the following tests: renal function (urea and creatinine) and hepatic function tests (alanine aminotransferase (ALT) and aspartate aminotransferase (AST)). For this analysis, the automated biochemical system (CELM SBA-200, Barueri, SP, Brazil) and Labtest^®^ commercial kits (Labtest Diagnóstica S.A., Lagoa Santa, MG, Brazil) were used, following the method described by the manufacturer.

### 2.5. ELISA

Two ELISA diagnostic kits were used to detect antibodies stimulated by immunotherapy (HSP70—Biogene Company, Brazil). For IgG Total analysis, sera were added at a 1:100 dilution. Peroxidase-conjugated rabbit anti-dog IgG antibodies were added at a 1:10,000 dilution for 30 min. The reaction was developed using TMB, and absorbance was measured on a Multiskan^®^ MCC 340 (Labsystems, Helsinki, Finland) automatic microplate reader at 450 nm. Cut-off was calculated according to each manufacturer’s determinations.

### 2.6. Isolation of Peripheral Blood Mononuclear Cells

All blood samples (20 mL) were collected in heparinized tubes from the 48 dogs. Peripheral blood mononuclear cells (PBMCs) were isolated as previously described [13]. Cells were counted in a Neubauer hemocytometer chamber to determine the number of monocytes or lymphocytes per milliliter. Isolated PBMC suspensions adjusted to 1 × 10 monocytes/well were added to 24-well plates (NUNC, Thermo Fisher Scientific Inc., Waltham, MA, USA). Cells were stimulated with 10 micrograms of soluble antigen from *L. infantum* and *L. amazonensis* (SLiAg and SLaAg) and maintained in culture for 72 h. Subsequently, the supernatant of each culture was collected and stored at −80 °C for cytokine measurement.

### 2.7. ELISA Cytokine Assay

Briefly, the plates were coated with anti-IFN-γ and anti-IL-10 mAb in PBS, pH 7.4, and incubated at 4 °C overnight. After blocking the wells using buffer containing PBS plus 0.05% (*v*/*v*) Tween 20 and 0.1% (*w*/*v*) BSA, supernatants were added to each well. Biotin-labeled mAb in incubation buffer was added to each well, and streptavidin–HRP was used as an enzyme. The reaction was developed using 3,3′,5,5′-tetramethylbenzidine substrate and stopped with 2.5 M H_2_SO_4_ solution. The plates were washed after each step of incubation using PBS plus 0.05% (*v*/*v*) Tween 20. Minimum sensitivity levels were 68 pg/mL for IFN-γ and 16 pg/mL for IL-10. All experiments were performed using 96-well plates (COSTAR, Corning Inc., Glendale, AZ, USA) and according to the instructions of R&D Systems. The reading was performed using a microplate automatic reader (Loccus^TM^, Cotia, São Paulo, Brazil) at a wavelength of 450 nm [15].

### 2.8. Parasite Load

The parasite loads were calculated by real-time PCR according to a method described elsewhere [16]. Skin biopsy was performed in the middle region of the left ear. A fragment was removed with the aid of sterile biopsy punch 5 mm in diameter (Punch for Biopsy^®^, Kolplast LTDA, São Paulo, São Paulo, Brazil) and stored in a freezer at −80 °C for further analysis of the parasite load. This material was collected before the start of immunotherapy at T0 and at all times after the end of the initial protocol. DNA was extracted from the skin using Nucleo Spin^®^ Tissue (Macherey-Nagel, Oerdt, France) according to the manufacturer’s instructions. The parasite loads were estimated using the following primers: forward, 5′TGTCGCTTGCAGACCAGATG 3′ and reverse, 5′GCATCGCAGGTGTGAGCAC 3′. These primers amplified a 90 bp fragment of a single-copy-DNA polymerase gene (DNA polα) from *L. infantum* (Gen-Bank accession number AF009147). PCR was carried out in a final volume of 10 μL containing 2.0 pmol of each primer, 2X SYBR^®^ Green PCR Master Mix (Applied Biosystems, Waitham, MA, USA), 4.0 μL of DNA with a final concentration of approximately 20 ng/mL, and ultrapure water. Reactions were processed and analyzed in an ABI Prism 7500 Sequence Detection System (SDS Applied Biosystems, Foster City, CA, USA). The following steps were programmed: an initial denaturation at 95 °C for 10 min followed by 40 cycles of denaturation at 95 °C for 15 s and annealing/extension at 60 °C for 1 min. Parasite quantification for each bone marrow and skin sample was calculated by interpolation of the standard curve included in the same run, performed in duplicate, and expressed as the number of *L. infantum* organisms per 20 ng of total DNA.

### 2.9. Statistical Analysis

The statistical analysis was performed with GraphPad Prism 9.0 software (Prism Software, Irvine, CA, USA). Data normality was assessed using the Kolmogorov–Smirnov test. The analyses were performed using a repeated-measures ANOVA. Differences were considered significant at *p* < 0.05.

## 3. Results

### 3.1. Immunotherapy Improves the Clinical Profile of Patients with CVL

Dogs in the initial stages that received the four-dose LaSap protocol showed improvement in clinical manifestations, especially in relation to exfoliative dermatitis and lymphadenopathy. The same was observed in the control group (La). In both groups, there was no recurrence of these clinical signs over the course of 12 months. In the group of patients in stages between 3 and 5, who received six doses, the LaSap group showed a more marked improvement compared to the La group. However, over the course of 12 months, some dogs returned to presenting the same clinical signs they had before starting treatment (Table 1 and Table 2).

### 3.2. LaSap Immunotherapy Stabilizes Most Staged Patients

The hematologic profile of patients in stages 1 and 2 remained stable throughout the treatment protocol, as well as throughout the four booster doses over 12 months. This profile was observed in both treatment groups, all remaining within the reference values (Table 3). On the other hand, in animals in more advanced stages (3 to 5), who received six doses of LaSap or La, it was observed that dogs in the La group presented a reduction in platelets (Table 4). In the LaSap group, most patients were able to maintain leukocyte levels within reference values. Of the seven dogs in stage 3, one progressed to stage 4 and six improved to stage 2. Of a total of four dogs that began the study in stage 4, two dogs progressed to level 5 in the last experimental trimester. One patient in stage 5 showed no improvement in his clinical condition (Table 3 and Table 4). Biochemical analyses indicated that only dogs in more advanced stages showed increased urea and creatinine levels in both treated groups.

### 3.3. Dogs May Exhibit Variable Antibody Levels Throughout Treatment with LaSap

**Figure 2:**
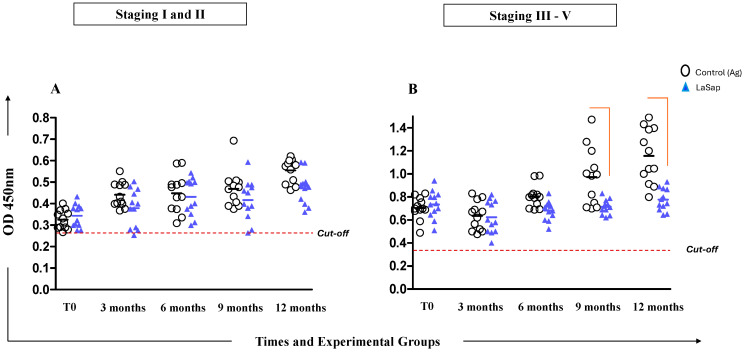
Enzyme-linked immunosorbent assay (ELISA) using recombinant HSP70 protein with sera from dogs naturally infected with *L. infantum* and treated with LaSap for 12 months after the initial protocol. (**A**) indicates the results from dogs in stages 1 and 2. (**B**) indicates the results from dogs in stages 3 to 5. The dashed lines indicate the cut-off; the connecting lines indicate statistical significance (*p* < 0.05).

Serological analyses performed using the HSP70 protein (commercial kit Biogene^®^), present in all *Leishmania* species, as a target, indicated that dogs in stages 1 and 2 were able to maintain antibody production at lower levels throughout the treatment. Dogs in stages between 3 and 5 already presented higher absorbances at time zero due to clinical conditions. However, the LaSap group was able to maintain production at lower values compared to the La group, especially at 9 and 12 months (Figure 2).

### 3.4. LaSap Reduces and Stabilizes Parasite Load in Most Treated Dogs

**Figure 3:**
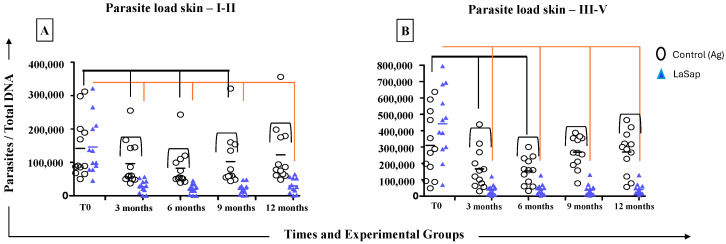
Analyses of parasite loads on the skin of 48 dogs naturally infected with *L. infantum* and treated with LaSap. (**A**) indicates the results in patients in stages 1 and 2. (**B**) indicates the results in patients in stages 3 to 5. The connecting lines indicate significant results (*p* < 0.05).

Dogs in stages 1 and 2 of the La group showed a significant reduction in parasite load on the skin up to 9 months after the end of the initial protocol (*p* = 0.04, 91,050 ± 57,520). On the other hand, dogs in the LaSap group remained with a lower parasite load up to 12 months (*p* = 0.0008, 84,800 ± 21,880). In addition, the parasite load of the LaSap group at 12 months was lower than that of the La group (*p* = 0.005, 90,000 ± 21,880) (Figure 3). In dogs in stages 3 and 5, the La group managed to reduce the parasite load up to 6 months after the end of the initial protocol (*p* = 0.023, 203,100 ± 83,260). The LaSap group maintained a lower load up to 12 months (*p* = 0.0001, 40,830 ± 33,620) compared to time zero (*p* = 0.0001, 442,400 ± 215,800). Similarly, there was a lower load compared to the La group (*p* = 0.0001, 269,700 ± 129,700) at 12 months.

In the bone marrow, dogs in the LaSap group in stages 1 and 2 maintained a lower parasite load at all times after the initial protocol, compared to time zero (T0 = 122,000; 3 months = 14,460; 6 months = 11,120; 9 months = 9745 and 12 months = 8726). At the last time, they also maintained a lower load than the La group (*p* = 0.006, 63,230 ± 8726). In the groups of dogs in stages between 3 and 5, there was a significant reduction in the load at times 9 (*p* = 0.043, 414,700 ± 139,500) and 12 months (*p* = 0.039, 536,200 ± 201,100).

### 3.5. LaSap Stimulates Higher Levels of IFN-γ in Dogs Naturally Infected with L. infantum

**Figure 4:**
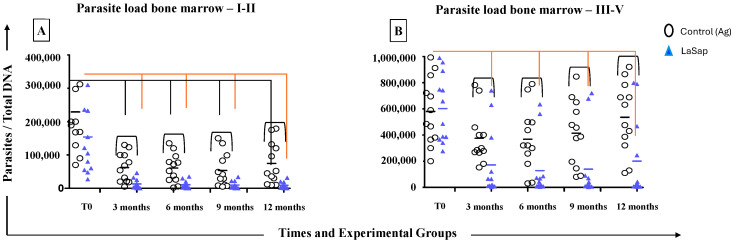
Analyses of parasite loads on the bone marrow of 48 dogs naturally infected with *L. infantum* and treated with LaSap. (**A**) indicates the results in patients in stages 1 and 2. (**B**) indicates the results in patients in stages 3 to 5. The connecting lines indicate significant results (*p* < 0.05).

**Figure 5:**
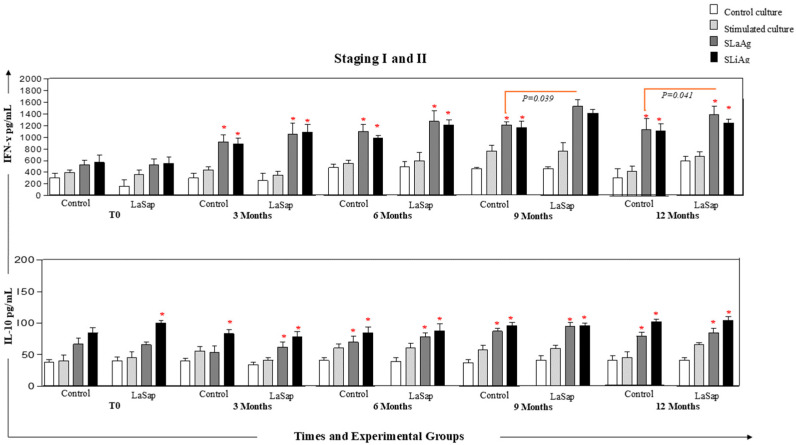
Analyses of IFN-γ and IL-10 cytokine levels from PBMC cultures of 24 dogs naturally infected with *L. infantum* and treated with LaSap over 12 months after the initial four-dose protocol (stages 1 and 2). Control culture 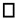, culture stimulated with LPS 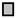, SLaAg 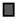 and SLiAg 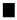. * and connecting lines indicate statistical differences within and outside the groups.

**Figure 6:**
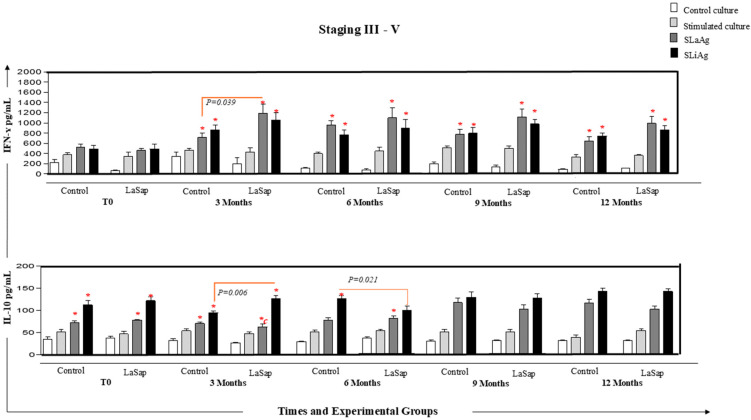
Analyses of IFN-γ and IL-10 cytokine levels from PBMC cultures of 24 dogs naturally infected with *L. infantum* and treated with LaSap over 12 months after the initial six-dose protocol (stages 3 to 5). Control culture 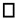, culture stimulated with LPS 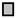, SLaAg 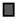 and SLiAg 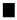. * and connecting lines indicate statistical differences within and outside the groups.

In dogs in stages 1 and 2, at all times analyzed, and in both the La and LaSap groups, there was greater production of *IFN-γ* in cultures stimulated with SLaAg and SLiAg compared to control cultures. However, at the 9-month time point (*p* = 0.041, 1.210, 1.533), there was greater production of the cytokine in the culture stimulated with SLaAg of the LaSap group compared to the La group. Regarding the production of IL-10, at all times, there was greater production of this cytokine in the stimulated cultures compared to the control cultures (Figure 4). A similar pattern was observed in dogs with stages between 3 and 5 (Figure 5). However, the levels of IL-10 were extremely low (*p* < 0.05) compared to IFN-g in the overall average (Figure 6).

## 4. Discussion

When it comes to CVL, there are different factors that directly impact the ability to control the transmission of *L. infantum*. Dogs are the main domestic reservoirs of the parasite in urban areas [17]. Euthanasia has not proven effective in most countries where this procedure has been used, in addition to the fact that dogs today have a completely different profile than in past decades, being treated as true members of the family [18]. Another factor that has emerged as even more aggravating is climate change, favoring the emergence of autochthonous cases in regions where there were no recorded cases, with the expansion of the vector [19,20,21] among many other factors.

Assuming that there are no drugs capable of achieving a sterilizing cure in dogs, CVL treatment aims to improve the patient’s clinical condition by reducing the parasite load; normalizing renal, hepatic, and hematologic profiles; preventing or delaying relapses; and reducing the transmission rate to the vector [22,23]. Until recently, the only drug licensed for the treatment of CVL in Brazil was miltefosine. However, after a few months, the treated dog experiences relapses, requiring further treatment with the same drug, generating parasite resistance [24]. The use of marbofloxacin as a leishmanicidal drug for the treatment of CVL was recently approved in Brazil. However, this is a concern among researchers due to the obvious issue of antibiotic resistance, especially because it is a protozoan, in addition to the transmission pressure in the country. In this context, this research group has been working for some years on an immunotherapy proposal containing *L. amazonensis* antigens plus saponin (LaSap) [14,16]. Previous results guided the present study, so that it was possible to verify the maintenance of dogs naturally infected with *L. infantum* for 12 months after the end of the initial protocol.

Dogs in stages 1 and 2 (Brasileish and Leishvet) [25,26] remained clinically stable throughout the study, with remission of the clinical signs presented before the start of treatment. Furthermore, this was observed in both treated groups, indicating that in these cases, the *L. amazonensis* antigen alone is sufficient. This is important because in clinical routine, there are patients who are sensitive to saponin [27] and who cannot receive the complete formulation. Dogs in stages 3 to 5 had greater difficulty in remaining stable over 12 months, especially patients in stage 4. Most dogs in stage 3 improved their scores, returning to stage 2. However, it is important to emphasize that animals in these stages already have kidney problems, directly impacting the patient’s clinical condition [28,29,30]. Another very important factor in the clinical management of the patient is the immunological condition itself, since there are dogs that are unable or partially capable of responding satisfactorily to the parasite [16,31,32].

Dogs in early stages treated with LaSap maintained lower IgG levels compared to animals in more advanced stages (Figure 2). There are several points to consider in this regard: (1) increased antibody levels are directly linked to clinical disease progression [33,34,35]; (2) immunotherapy also stimulates antibodies, but the ELISA kit used in this study did not allow us to distinguish between antibodies stimulated by infection and immunotherapy, as the HSP70 protein in the kit is present throughout the Leishmania genus. However, dogs that better control the infection produce fewer antibodies [24,36]. Over the course of 12 months after the initial treatment, both in the skin and in the bone marrow, dogs treated with LaSap presented lower parasite loads compared to the La group in all stages analyzed, indicating the importance of saponin in immunotherapy. This was also verified in previous studies [13,14]; however, with different treatment protocols than the current study. Improvement in parasitological indices was also obtained in other studies with different formulations, including nutraceuticals [37,38,39]. In this context, it was evident that immunotherapy with LaSap stimulated higher levels of IFN-γ in relation to IL-10 over a year after the initial treatment, maintaining a booster dose every three months. IFN-γ plays an important role in the activation of macrophages and consequent reduction in the parasite load [40,41,42,43].

**Figure 7:**
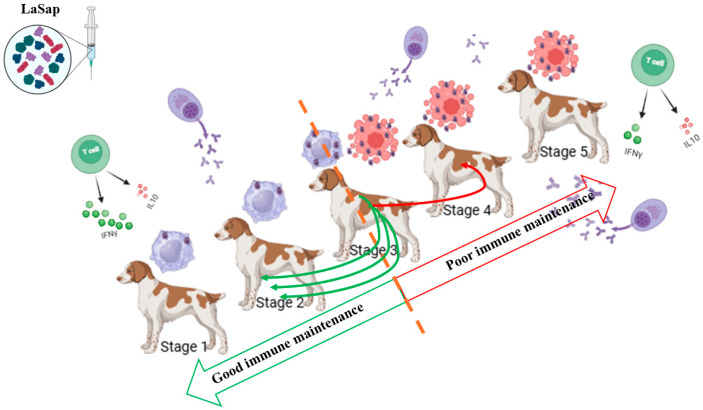
Overview of dogs in different clinical stages of visceral leishmaniasis (Brasileish and Leishvet) treated with LaSap. Dogs in stages 1 and 2 present better results with monotherapy, including clinical maintenance of the patient, with lower production of IgG antibodies, greater cellular response, and more incisive reduction of the parasite load (skin and bone marrow). On the other hand, dogs in more advanced stages, especially those in stage 4, present a poor response profile using LaSap alone. Dogs in stage 3 play a key role in the progression to level 4 or regression to level 2 (dashed orange line), and this seems to be more related to the individual immune response profile of the patient. However, most dogs in this stage can return to stage 2 if there is clinical monitoring by the veterinarian, especially with monitoring of the renal profile.

Considering that CVL is a multifaceted and complex disease in which each patient responds very uniquely to the same treatment, it is reasonable to infer that dogs in different stages should receive different immunotherapeutic treatment protocols with LaSap. This was most evident when we found that stage 3 of the disease is the threshold between keeping the patient stable and returning to stage 2 or progressing to stage 4 (Figure 7). Dogs in more critical conditions received an initial protocol of six doses, the first two administered seven days apart, as a way of generating a greater stimulus in a shorter time. Thereafter, the doses were applied at longer intervals (Figure 1). In contrast, dogs in earlier stages can receive fewer doses at the same interval, since they are in better clinical condition. Furthermore, it is clear that dogs in critical conditions, especially those with advanced renal disease, cannot be maintained with immunotherapy alone. However, based on the results of this study, dogs in stages 2 and 3, depending on the response conditions, can be maintained stabilized with LaSap alone for 12 months (Figure 7).

## 5. Conclusions

In addition to demonstrating safety, the strategy of using different immunotherapeutic protocols with LaSap according to the patient’s clinical staging appears to be appropriate, considering the results obtained in this clinical study.

## Figures and Tables

**Table 1:** Relationship between dogs naturally infected with *L. infantum* (stages 1 and 2) and treated with LaSap, with clinical signs present at different analyses times over 12 months after the initial 4-dose protocol. Time zero (before starting the protocol), T1 (3 months after the protocol), T2 (6 months after the protocol), T3 (9 months after the protocol), and T4 (12 months after the protocol).

Clinical Signs Staging 1 and 2	0 Time		3 Months		6 Months		9 Months		12 Months	
	La	LaSap	La	LaSap	La	LaSap	La	LaSap	La	LaSap
Alopecia/exfoliative dermatitis	10/12	9/12	0	0	0	0	0	0	0	0
Lymphadenopathy	8/12	7/12	0	0	0	0	0	0	0	0
Lethargy										
Cachexia										
Epistaxis										
Vasculitis										
Arthritis										

**Table 2:** Relationship between dogs naturally infected with *L. infantum* (stages 3 to 5) and treated with LaSap, with clinical signs present at different analyses times over 12 months after the initial six-dose protocol. Time zero (before starting the protocol), T1 (3 months after the protocol), T2 (6 months after the protocol), T3 (9 months after the protocol) and T4 (12 months after the protocol).

Clinical Signs Staging 3–5	0 Time		3 Months		6 Months		9 Months		12 Months	
	La	LaSap	La	LaSap	La	LaSap	La	LaSap	La	LaSap
Alopecia/exfoliative dermatitis	8/12	10/12	0/12	0/12	2/12	0	4/12	1/12	5/12	1/12
Lymphadenopathy	10/12	12/12	4/12	0/12	4/12	1/12	5/12	1/12	5/12	2/12
Lethargy	4/12	5/12	0/12	0/12	1/12	0/12	1/12	0/12	2/12	1/12
Cachexia	3/12	4/12	0/12	0/12	1/12	0/12	1/12	0/12	1/12	0/12
Epistaxis	2/12	2/12	0/12	0/12	1/12	0/12	2/12	0/12	2/12	2/12
Vasculitis	6/12	6/12	3/12	0/12	3/12	0/12	4/12	1/12	5/12	2/12
Arthritis	1/12	2/12	0/12	0/12	1/12	0/12	1/12	1/12	1/12	1/12

**Table 3:** Hematological and biochemical renal and hepatic results of the La and LaSap groups in stages 1 and 2 of CVL at different analysis times over 12 months after the initial four-dose protocol. Time zero (before starting the protocol), T1 (3 months after the protocol), T2 (6 months after the protocol), T3 (9 months after the protocol) and T4 (12 months after the protocol).

Clinical Signs Staging 1–2	0 Time		3 Months		6 Months		9 Months		12 Months		Reference
	La	LaSap	La	LaSap	La	LaSap	La	LaSap	La	LaSap	
Total leukocytes	12,401	8068	12,891	8914	8819	10,463	10,250	10,604	8234	9214	6000–17,000 mm^3^
Rods	0	0	0	0	0	0	0	0	0	0	0–300
Segmented	7875	4228	7893	5495	4704	5580	4984	5833	4680	5022	3000–11,500
Lymphocytes	3570	2579	3891	2858	3072	3900	3921	3682	2954	3240	1000–4800
Monocytes	767	317	866	371	800	843	1024	810	498	672	150–1350
Eosinophils	222	186	239	188	243	140	321	279	102	280	100–1250
Platelets	346,000	315,000	345,000	329,000	322,000	354,000	319,000	377,000	320,000	350,000	200,000–500,000 mm^3^
AST	45	52	37	40	58	44	39	31	64	48	10–88 U/L
ALT	50	42	33	28	57	39	61	46	52	37	10–88 U/L
Urea	22	37	29	23	38	42	30	39	43	38	20–50 mg/dL
Creatinine	0.9	1.0	1.2	1.1	0.89	1.0	1.1	1.2	1.0	0.8	0.5–1.4 mg/dL

**Table 4:** Hematological and biochemical renal and hepatic results of the La and LaSap groups in stages 3 to 5 of CVL at different analyses times over 12 months after the initial six-dose protocol. Time zero (before starting the protocol), T1 (3 months after the protocol), T2 (6 months after the protocol), T3 (9 months after the protocol) and T4 (12 months after the protocol).

Clinical Signs Staging 3–5	0 Time		3 Months		6 Months		9 Months		12 Months		Reference
	La	LaSap	La	LaSap	La	LaSap	La	LaSap	La	LaSap	
Total leukocytes	7420	7968	9370	10,311	7250	9230	7821	10,590	6294	8760	6000–17,000 mm^3^
Rods	0	0	0	0	0	0	0	0	0	0	0–300
Segmented	4823	5557	6465	7320	4640	6398	4849	7200	4405	5606	3000–11,500
Lymphocytes	1946	1788	2010	2410	1620	1890	1972	2287	1200	2468	1000–4800
Monocytes	319	388	621	300	660	785	840	847	422	398	150–1350
Eosinophils	332	238	274	281	330	157	160	256	267	288	100–1250
Platelets	240,000	276,000	350,000	392,000	228,000	312,000	196,000	260,000	182,000	220,000	200,000–500,000 mm^3^
AST	65	74	61	65	69	57	76	60	85	63	10–88 U/L
ALT	72	69	59	56	66	55	75	67	73	65	10–88 U/L
Urea	50	52	36	38	40	35	47	44	55	50	20–50 mg/dL
Creatinine	1.6	1.7	1.3	1.1	1.4	1.2	1.4	1.1	1.5	1.4	0.5–1.4 mg/dL

## Data Availability

The data presented in this study are available on request from the corresponding author.

## Abbreviations

The following abbreviations are used in this manuscript:

CVL	Canine visceral leishmaniasis
LaSap	*L. amazonensis* antigens plus saponin
WHO	World Health Organization
VL	Visceral leishmaniasis
La	*L. amazonensis*
ALT	Alanine aminotransferase
AST	Aspartate aminotransferase
PBMC	Peripheral blood mononuclear cells
SLiAg	Micrograms of soluble *L. infantum* antigen
SLaAg	Micrograms of soluble *L amazonensis* antigen
DNA polα	DNA polymerase
